# Pharmacokinetics and pharmacogenetics of Gemcitabine as a mainstay in adult and pediatric oncology: an EORTC-PAMM perspective

**DOI:** 10.1007/s00280-016-3003-0

**Published:** 2016-03-23

**Authors:** Joseph Ciccolini, Cindy Serdjebi, Godefridus J. Peters, Elisa Giovannetti

**Affiliations:** Pharmacokinetics Unit, SMARTc, Inserm S_911 CRO2, Aix Marseille University, Marseille, France; Department of Medical Oncology, VUmc, Amsterdam, The Netherlands; Cancer Pharmacology Lab, AIRC/Start-Up Unit, University of Pisa, Pisa, Italy

**Keywords:** Gemcitabine, 2′,2′-difluoro-2′-deoxyuridine, Cytidine deaminase, Polymorphisms, Pharmacogenetics, Gemcitabine prodrugs

## Abstract

Gemcitabine is an antimetabolite ranking among the most prescribed anticancer drugs worldwide. This nucleoside analog exerts its antiproliferative action after tumoral conversion into active triphosphorylated nucleotides interfering with DNA synthesis and targeting ribonucleotide reductase. Gemcitabine is a mainstay for treating pancreatic and lung cancers, alone or in combination with several cytotoxic drugs (nab-paclitaxel, cisplatin and oxaliplatin), and is an option in a variety of other solid or hematological cancers. Several determinants of response have been identified with gemcitabine, i.e., membrane transporters, activating and inactivating enzymes at the tumor level, or Hedgehog signaling pathway. More recent studies have investigated how germinal genetic polymorphisms affecting cytidine deaminase, the enzyme responsible for the liver disposition of gemcitabine, could act as well as a marker for clinical outcome (i.e., toxicity, efficacy) at the bedside. Besides, constant efforts have been made to develop alternative chemical derivatives or encapsulated forms of gemcitabine, as an attempt to improve its metabolism and pharmacokinetics profile. Overall, gemcitabine is a drug paradigmatic for constant searches of the scientific community to improve its administration through the development of personalized medicine in oncology.

## Introduction

Among the pyrimidine analogs, gemcitabine (2′,2′-difluorodeoxycytidine, dFdC; Gemzar^®^) is one the most widely used drugs in clinical oncology and ranked the third anticancer agent prescribed worldwide. It is a cytidine analog, where two fluorine atoms have replaced the hydroxyl on the ribose. In particular, gemcitabine is a mainstay in pancreatic adenocarcinoma [1, 2] and is widely prescribed to treat a variety of other solid tumors such as breast, ovarian, bladder or non-small-cell lung (NSCLC) cancers [3, 4]. In addition to solid tumors, gemcitabine is indicated as well in several hematological disorders such as acute leukemia [5]. Beyond adult patients, gemcitabine can be an attractive option in pediatric cancers because its toxic profile is usually considered as mild as compared with other cytotoxic drugs.

**Fig. 1:**
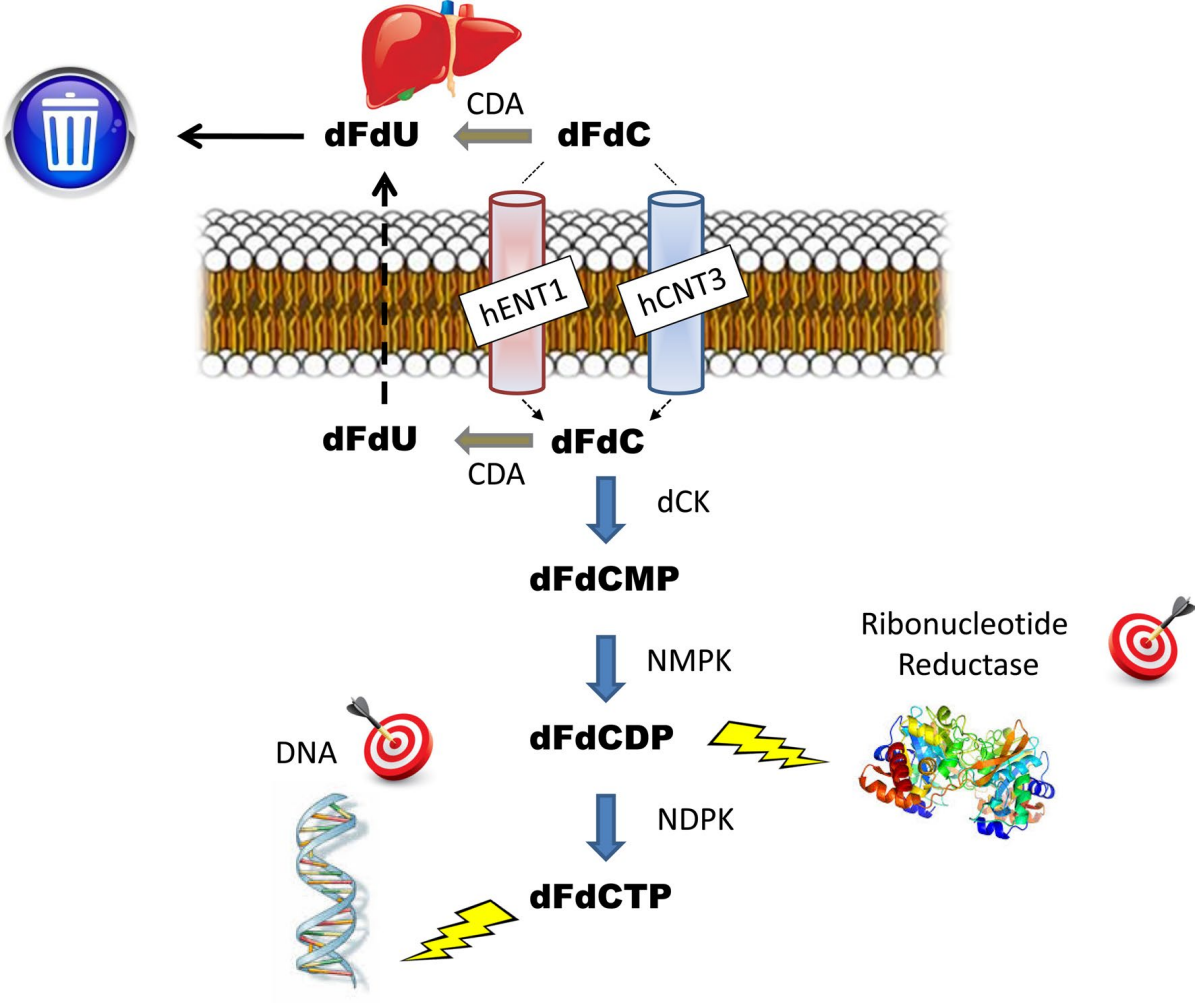
Gemcitabine (dFdC) patterns and mechanisms of action. *CDA* cytidine deaminase, *dCK* deoxycytidine kinase, *NMPK* nucleotide monophosphate kinase, *NDPK* nucleotide diphosphate kinase, *hENT1* human equilibrative nucleoside transporter-1, *hCNT3* human concentrative nucleoside transporter-3. In cancer cells, genetic polymorphisms affecting membrane transporters, activating and deactivating enzymes and pharmacological targets such as ribonucleotide reductase, are all associated with treatment efficacy

After administration and taken up by the cancer cell, gemcitabine undergoes an initial phosphorylation by deoxycytidine kinase (dCK) and to a lower extent by the extra-mitochondrial thymidine kinase 2, followed by a series of phosphorylation steps in order to be incorporated into both DNA and RNA as its active phosphorylated form gemcitabine triphosphate (dFdCTP) [6]. Additionally, gemcitabine diphosphate (dFdCDP) inhibits ribonucleotide reductase (RR), an enzyme in the nucleotide pathway critical for the cancer cell to manage its pools of deoxynucleotides. The clearance of gemcitabine is mostly driven by rapid and extensive inactivation by cytidine deaminase (CDA) to its primary metabolite 2′,2′-difluoro-deoxyuridine (dFdU); CDA is expressed ubiquitously at high levels in both plasma and the liver, [7]. A 24-h hepatic artery infusion of gemcitabine to the liver underlined the important role for liver CDA-mediated catabolism to dFdU, since the Cmax and area-under-curve of dFdU were similar for the hepatic artery infusion and a 24-h intravenous infusion of gemcitabine, while gemcitabine plasma levels were much lower after the hepatic artery infusion [8]. Figure 1 briefly summarizes these main steps of gemcitabine metabolism and mechanisms of action.

Because gemcitabine is the backbone of numerous regimens, several studies have tried to identify molecular or genetic determinants of response, both at the somatic and the constitutional levels [9]. In addition, recent efforts have been made to improve the metabolism and pharmacokinetics (DM-PK) profile of gemcitabine, creating novel chemical derivatives, prodrugs or nanomedicine forms [10].

## Gemcitabine pharmacokinetics and pharmacodynamics

Because of its hydrophilic nature, gemcitabine does not readily cross the membrane by diffusion, and it is transported into the cells by membrane nucleoside transporters [11]. Following cellular uptake, gemcitabine is phosphorylated to its active diphosphate (dFdCDP) and triphosphate (dFdCTP) metabolites, which inhibit RR and DNA synthesis, respectively [12]. dCK is the rate-limiting enzyme in the biotransformation of nucleoside analogs, and several studies have suggested that dCK is a limiting factor for gemcitabine activity, because its deficiency/modulation is critically involved in acquired resistance in different in vitro models [13, 14]. Moreover, pretreatment dCK expression level could be used as a predictive parameter of tumor sensitivity, as observed with a clear correlation between dCK activity and gemcitabine sensitivity in tumor cells and xenografts [15].

The dynamics of dFdCDP and dFdCTP formation and activity in vivo are complex; dFdCTP is incorporated into DNA followed by one or more deoxynucleotides masking gemcitabine and preventing DNA repair by 3′5′-exonuclease activity, a process designated as “masked DNA chain termination” [16]. This causes an S-phase-specific cell cycle arrest and programmed cell death. dFdCTP is also incorporated into RNA, thus inhibiting RNA synthesis [17], while dFdCDP inhibits RR, inducing a depletion of the cellular pool of deoxynucleoside triphosphates, and blocks the de novo DNA synthesis pathway [18].

However, only a proportion of gemcitabine is converted into the active di- or triphosphate forms. The majority of gemcitabine is rapidly inactivated in the liver and to a lesser extent in blood by deamination into dFdU, through a reaction catalyzed by CDA. Additionally, 10 % of unchanged gemcitabine can undergo renal filtration, and within 1 week, more than 90 % of the injected dose is usually recovered in the urine, either as parent gemcitabine (1 %) or dFdU (99 %) [19]. In addition, the formation of dFdCTP and dFdCDP from dFdCMP is reduced through deamination of dFdCMP to 2′,2′-difluorodeoxyuridine monophosphate (dFdUMP) by dCMP deaminase. Notably, an elevated concentration of dFdCTP inhibits dCMP deaminase, determining a “self-potentiation” of the drug activity [20], which is also caused by the increase in dFdCTP accumulation induced by dFdU in a time-dependent manner [21]. dFdCTP also inhibits CTP synthetase, affecting RNA synthesis by depletion of CTP, while the latter decreases dCTP synthesis [22, 23]. Finally, a recent study demonstrated that gemcitabine can inhibit the enzyme thymidylate synthase presumably through the phosphorylated metabolite dFdUMP. Inhibition of this enzyme enhances the mis-incorporation of 2′-deoxyuridine into DNA, causing indirect damage [24].

A considerable inter-patient variability has been described in gemcitabine accumulation, and the pharmacokinetics of gemcitabine and its main metabolite dFdU in plasma have been evaluated in multiple studies. Gemcitabine plasma concentrations generally reach a plateau after 15–30 min during the standard 30 min infusion protocol. Linear pharmacokinetics have been described over the range 40–3650 mg/m^2^, and nonlinear pharmacokinetics at higher doses [19, 25, 26]. Mean gemcitabine peak plasma concentrations ranged from 24 μM at 800 mg/m^2^ [27] to 32 μM at 1000 mg/m^2^ [28], around 53–70 μM at a dose of 1250 mg/m^2^ [29, 30], 68–79 µM at 2350 mg/m^2^ and between 320 and 512 µM at the MTD of 5700 mg/m^2^ [19]. Up to at least at gemcitabine 1250 mg/m^2^, deamination was linear with mean plasma dFdU concentrations being 1.25 times higher as compared to dFdU levels using gemcitabine 1000 mg/m^2^. Linearity was lost at doses higher than 3650 mg/m^2^ [19]. The clearance of gemcitabine in the plasma is also rapid (i.e., T1/2 of 5–20 min). More than 75 % of gemcitabine is metabolised to dFdU and excreted in the urine in the first 24 h [19]. This clearance is independent of dose over the linear range (i.e., up to 3650 mg/m^2^), but proportional to creatinine clearance. At the highest doses, the clearance was lower; moreover, the clearance was 1.5-fold higher in men (8.6 l/m^2^) compared to women (5.7 l/m^2^) [19]. The pharmacokinetic elimination half-life for dFdU varies between 2 and 24 h, and it is still present systemically in concentrations greater than 1 μM up to 1 week after dosing [31]. Of note, since dFdU is not protein bound, its plasma concentration, up to 460 μM [19], depending on the dose administered, is freely available. These concentrations are cytotoxic [20, 32] and could have significant implications in the clinical use of gemcitabine alone or in combination with other therapies, such as radiation [33], since dFdU has a radiosensitizing effect by itself.

Since gemcitabine is often given in combination with other cytotoxic and targeted drugs, the effect of combination therapy on the pharmacokinetics has been investigated in several clinical studies, since theoretically co-medication can affect both drug metabolism and elimination. However, in the most widely used combination with cisplatin or paclitaxel, no evidence was found for an effect of these drugs (as well as oxaliplatin and carboplatin) on both gemcitabine and dFdU pharmacokinetics, investigated within the same patients and between patients [27, 28, 30, 34, 35]. Similarly, the proteasome inhibitor bortezomib and the farnesyltransferase inhibitor SCH66336 did not affect the pharmacokinetics of gemcitabine or dFdU, alone or in the combination with cisplatin [36, 37]. Moreover, no effect of the VEGFR inhibitor SU5416 was observed [29], while the EGFR inhibitor gefitinib tended to increase the exposure to gemcitabine [38]. However, the other EGFR inhibitor erlotinib did not affect pharmacokinetics of the gemcitabine prodrug LY2334737 itself or of gemcitabine and dFdU [39]. Hence, from the point of view of pharmacokinetics, it can be concluded that in general gemcitabine can safely be combined with other drugs, both other cytotoxics and novel targeted drugs. Naturally, this does not exclude that gemcitabine affects the mechanism of action of other drugs or that these other drugs affect intracellular metabolism of gemcitabine. Two examples include the potentiation by gemcitabine of cisplatin adduct formation and the selective effect of bortezomib on intracellular gemcitabine activation [27, 40].

Less data are available on the pharmacokinetics of dFdCTP, which should be measured with more sensitive LCMS assays [41]. However, several studies demonstrated that cells exposed to gemcitabine have saturable accumulation of the dFdCTP, and the optimal plasma concentration of gemcitabine that maximized the rate of formation of dFdCTP was approximately 20 μmol/l [42, 43]. This is accompanied by a change in the pattern of elimination with monophasic elimination at low concentrations and biphasic elimination described after the threshold has been reached.

Since the optimal intracellular accumulation of dFdCTP was achieved with dose rates of 10 mg/m^2^/min [25, 43], a number of phase I trials have explored the possibility to prolong the duration of infusion time, while other trials escalated both the dose and infusion duration [25, 44, 45]. The rationale for prolonged dosing received a major boost when a randomised phase II trial in a clinically relevant scenario (pancreatic cancer) demonstrated that prolonged infusion at a rate of 10 mg/m^2^/min, compared to the standard dosing regimen with 30 min infusion, was associated with increased accumulation of dFdCTP, as well as with a significant increase in response rate and a trend for increased survival [46]. Similar trials in different tumor types confirmed the pharmacokinetic finding, but were underpowered to demonstrate survival differences [47]. Unfortunately, a large phase III study in pancreatic cancer showed that the pharmacological advantage failed to translate into a significant survival advantage [34].

Collectively, these clinical studies indicate that the anti-tumor effect of gemcitabine is schedule dependent and that lower doses can be efficacious. Therefore, it could be advantageous to deliver gemcitabine in a manner where it can achieve prolonged systemic exposure, good efficacy with lower toxicity along with added flexibility of administration and greater patient convenience, such as using an oral formulation [48]. However, administering gemcitabine orally to patients has been limited by low oral bioavailability, high first-pass clearance, variable systemic exposures during dose escalation studies and observation of gastrointestinal toxicity including nausea, vomiting and diarrhea.

## Dysregulation at the germinal level: pharmacogenetics of gemcitabine

Factors extracted from either clinical or pathological data such as age, performance status, comorbidity and disease stage or grade provide a crude discrimination of prognosis, but are often not predictive and not helpful for the choice of the best chemotherapeutic regimen for a given patient. Novel approaches to stratify patient’s prognosis or toxicity may be offered by pharmacogenetic analyses of selected candidate polymorphisms that could influence the expression of genes involved in drug metabolic pathways.

Historically, pharmacogenetics is indeed defined as the study of germline mutations (e.g., single-nucleotide polymorphisms affecting genes coding for enzymes responsible for drug pharmacokinetics), whereas pharmacogenomics refers to the role of both acquired and inherited genetic differences in relation to drug behavior through a systematic examination of genes, gene products and inter- and intra-individual variation in gene expression and function using new genomic technologies [49]. However, in oncology, pharmacogenetics is often considered as concerning the individual patient’s features and pharmacogenomics as those of the tumor.

### CDA deregulations and clinical outcome

Gemcitabine is primarily detoxified in the liver by CDA into dFdU, with a Km of approximatively 96 µM [50, 51]. Usually, 90 % of gemcitabine is detoxified by CDA, and variations in enzymatic activity impact greatly on drug pharmacokinetics and pharmacodynamics. Mice with impaired CDA displayed sharp overexposure to the drug with subsequent unrecoverable hematological toxicities [52], thus highlighting the correlation between CDA deficiency, overexposure to gemcitabine and increased risk of severe toxicities. Indeed, a variety of studies and case reports have found a correlation between CDA deficiency syndrome and an increase in severe hematological toxicities in patients undergoing gemcitabine-based therapy [52, 53]. Of note, the first-ever reported case of toxic death related to CDA deficiency in an ovarian cancer patient treated with the gemcitabine—carboplatin was published in 2007 [53]. Profound functional deficiency was retrospectively evidenced, with heterozygous CDA*2 genotype. Subsequent genetic investigations revealed a new intronic mutation (i.e., 154 + 37G>A) on the CDA gene, likely to have caused the lethal toxicities [54]. Of note, other studies have shown that patients with lower CDA activity also tend to display higher response rates and better survival [55, 56]. On the contrary, it has been observed that about 15 % of the Caucasian adult population display CDA activities significantly higher than the median values of adult populations (i.e., over 6 U/mg), making them prone to therapeutic failure because most of standard dosing of gemcitabine will be metabolized in the liver before it even reaches the tumor tissues [52]. A pilot study involving 40 patients treated by gemcitabine-based regimens for pancreatic cancer confirmed that patients displaying CDA ultrametabolizer phenotype were fivefold more at risk to have a progressive disease than patients with normal CDA status [57]. As expected, these patients had milder toxicities than patients with normal or lower CDA activity, an observation completely in line with previous reports about CDA and gemcitabine-related toxicities. Overall, all these studies, conducted by independent groups and involving patients treated with gemcitabine used alone or in a combination for a variety of settings, demonstrate how CDA status greatly affects clinical outcome in patients undergoing gemcitabine-based treatments.

### CDA genetic polymorphisms

CDA is coded by the 4-exons gene CDA located on the first pair of chromosomes (1p36.2-p35). CDA is formed by four identical subunits, all presenting a zinc atom in the active site. It is mostly expressed in liver and placenta, but high levels of CDA are also expressed in mature neutrophils and erythrocytes [58]. CDA is responsible for the physiological deamination of cytidine and 2′-deoxycytidine into uridine and 2′-deoxyuridine, respectively. Because a wide inter-patient variability has been observed with CDA, numerous studies have been undertaken to screen for possible mutations and polymorphisms affecting the *CDA* gene since the mid-70s, both in germinal cells and in cancer cells [59–62]. As of today, up to 1000 genetic variations affecting CDA have been described. The most studied polymorphisms are the two non-synonymous 79A>C (rs2072671) and 208G>A (rs60369023) substitutions and the synonymous 435C>T (rs1048977) variant [63–66]. Beside these polymorphisms affecting coding regions, many other mutations of the promoter region such as the −31delC deletion (rs3215400) or −92A>G (rs602950), or in intronic regions such as the 154 + 37G>A polymorphism (rs12059454) have been described [54, 67–70]. All these genetic variations lead to inconsistent and sometimes conflictual results in term of resulting phenotypic status [69, 70], as reported in the Table 1.

**Table 1:** CDA polymorphisms and their differential functional and clinical effects

Polymorphism	Ethnic variation	Functional effects	Clinical effects
−897 C>A	Reported in Asians, Africans and Caucasians [71, 85, 111, 112]	Different activity (increased or reduced) among haplotypes [59]	Unknown
−451 C>T			Severe capecitabine-induced hand-foot syndrome [71] and survival [66]
−92 A>G			Unknown
−111 C>T	Reported in Asians and Africans, unknown in Caucasians [85, 111, 112]	Unknown	Gemcitabine induced neutropenia [113]
79 A>C (Lys27Gln)	Reported in Asians, Africans and Caucasians [85, 112]	Reduced [61] or unaltered [63] or increased [55, 75, 76] activity	Reduced survival and toxicity in PDAC and NSCLC patients [54, 55, 76], severe neutropenia in leukemia patients [78] treated with gemcitabine
208 G>A (Ala70Thr)	Reported in Asians, not in Caucasians [78, 112]	Reduced activity [73]	Gemcitabine induced severe (life-threatening) toxicities [73, 74, 83, 85]
435 C>T (Thr145Thr)	Reported in Asians, Africans and Caucasians [112]	Unaltered activity [55]	CDA 435 C/C associated with better response and progression-free survival while C/T with a significantly increased risk of non-hematological toxicity [62]

Indeed, the large inter-individual variability reported with CDA activity is only partly explained by the genetic background. In addition, because more than 1000 genetic variations have been evidenced, SNP-candidate studies are probably underpowered strategies, yielding conflictual data [51, 55, 64, 68, 71, 72]. For instance, the 79A>C polymorphism (i.e., CDA*2) leads to lysine to glutamine permutation in position 27, with no impact on the catalytic site eventually, but other factors might play a role. Of note, ethnicity plays a crucial role in the allelic frequencies of this variant because minor allele frequency (MAF) ranges from 10 % in African population, 15 % in Asian population, but up to 35 % in Caucasians [73, 74]. The phenotypic impact of this allelic variant and its consequence in the clinical outcome in patients treated with nucleoside analogs remain controversial: a decrease in CDA activity has been measured for the Lys^27^Lys variant [75, 76], whereas other studies suggest no variation [52, 77] or lower activity for the Gln^27^Gln variant [78, 79]. These differences may be partly explained by variations in study design such as patient selection, ethnicity and treatment regimens [80–82]. A pivotal study has recently been published, collecting data about CDA catalytic activity according to substrates, both natural and synthetic [83]. This biochemical study, inspired from a previous work in 2012 by Baker and collaborators [79], highlights that catalytic efficiency of CDA enzyme is dependent on the genetic sequence encoding for the protein, but also on the drugs used as substrates. Surprisingly, an increase in CDA catalytic efficiency was observed for the CDA^27^Gln protein with natural cytidine analogs and cytarabine, but surprisingly a decrease was found for other substrates such as 5-azacytidine, 6-azacytidine and fazarabine. This is in agreement with the results of the study by Giovannetti et al. [75], in which CDA^27^Gln activity was investigated with gemcitabine, with a decrease in deaminase activity being observed with this polymorphism.

In addition to the CDA*2 allelic variant, another polymorphism has been studied extensively: CDA*3, resulting from the substitution of alanine to threonine in position 70 because of the 208G>A SNP in the coding sequence. With the CDA*3 variant, impact on CDA phenotype is more univocal because researchers all agree that a decrease in CDA activity is found for the protein encoded by 208 A/A variant [68, 74, 83]. Indeed, deaminase activity was found to be 100-fold lower than normal CDA with respect to all tested drugs with this allelic variant [57]. Of note and unlike CDA*2, which is found in every population, but in different proportions, CDA*3 has never been detected in Caucasian populations, but is only found in Africans and Asians. To date, the clinical impact of CDA*3 genotype in patients treated with gemcitabine has been repeatedly reported in Japanese patients only [74, 83, 84]. In addition, two studies have aimed at establishing the respective MAF of 79A>C, 208G>A and 435C>T in both Asian and Caucasian populations [67, 85]. Few differences were observed in MAF of 435C>T allelic variant when comparing these two ethnicities, whereas discrepancies were evidenced for the 79A>C and 208G>A variants. For the CDA*2 allelic variant, twice as many individuals carry a wild-type genotype with a lower incidence of C/C genotype in Asians, as compared with white people. The discrepancy is more marked for the rs60369023 variant because no individual, whether in African-Americans, Chinese-Americans, or Caucasian-Americans, was carrying the minor allele A. Only 11 patients were heterozygous among over 400 Korean and Japanese patients, and none of them was found to be homozygous for the CDA*3 variant [85]. These data confirm that screening for 208G>A single-nucleotide polymorphism has a clinical, yet limited, meaning in Asian or more significantly in Japanese populations only. As mentioned above, numerous other genetic variations have been identified, but no study has established a clear link between a given genotype and the resulting phenotype yet, apart for the −31delC variant (rs532545), a CDA promoter deletion possibly resulting in an amplification of the CDA gene with functional (i.e., ultrametabolizer phenotype) impact eventually [71]. These data call for more sophisticated multigenic or haplotype-based studies to establish a clear genotype-to-phenotype relationship with CDA and gemcitabine.

## Dysregulation at the tumor level: pharmacogenomics of gemcitabine

Several determinants for efficacy have been identified with gemcitabine at the tumor level. Because gemcitabine requires facilitated transport for cellular uptake [11], several studies evaluated the expression levels of the plasma membrane human equilibrative nucleoside transporter-1 (hENT1) and human concentrative nucleoside transporter-3 (hCNT3), showing their prognostic and predictive roles of drug activity in patients undergoing gemcitabine-based regimen [86–89]. Higher uptake in cancer cells with high levels of both transporters could explain the marked increase in disease-free survival and overall survival observed in pancreatic cancer patients administered with gemcitabine. Of note, several polymorphisms affecting the genes coding for hENT1 and hCNT3 have been described. These polymorphisms might impact protein expression, but the functional and clinical significance of these polymorphisms have yet to be defined [10].

Other determinants for response at the tumor level include the expression of dCK, the rate-limiting enzyme activating the prodrug gemcitabine to active nucleotides [90], deoxycytidylate deaminase that metabolizes active phosphorylated nucleotides into inactive metabolites [6] and RR that is one of the gemcitabine targets [91]. These genes have many polymorphisms, which could impact on drug efficacy, but the relevance of all these markers has not been fully confirmed at the bedside. Large prospective control studies are necessary to confirm the role of these determinants in response to gemcitabine.

However, the candidate-gene approach used in most of these studies cannot establish if a positive association is due to linkage with untyped functional variant alleles or due to intragene interaction. Drug efficacy and toxicity may also be influenced by other genes and pathways, which will be undetected by single-polymorphism analysis. Therefore, alternative approaches through the broader application of new genomic technologies might be necessary to identify novels biomarkers of gemcitabine efficacy and toxicity, and to bring us closer to tailor-made therapy for individual patients [92]. Li et al. [93] used such a method to identify novel genes involved in gemcitabine metabolism. After analyzing data with 26,653 probe sets, the researchers identified 15 genes where mRNA expression correlated with cytidine analog sensitivity and, from there, selected FKBP5 for further functional variation. FKBP5 is a gene involved in steroid receptor mutation and is a binding partner for rapamycin, suggesting that it may have a role in the apoptosis pathway. Overexpression of FKBP5 correlated with increased gemcitabine chemosensitivity. Using FKBP5 siRNA-treated cells, the researchers showed a decrease in downstream enzyme caspase-3/7 activity, confirming that the activation of the apoptotic pathway was affected by the downregulation of FKBP5 expression.

Recently, it has also been shown that tumor blood perfusion and vessels’ density could be associated with response to drug-based therapy, probably in relation with a drug delivery issue. In this respect, the Hedgehog signaling pathway has been suggested as being a new determinant of response with gemcitabine, since it is associated with production of a desmoplastic tumor stroma and a reduced tumoral perfusion eventually, probably through the expression of Gli family transcriptor factors. As a result, Hedgehog signaling could prevent at least partly gemcitabine to be delivered to tumors [94, 95]. This rising concern in both experimental and clinical oncology about the role Hedgehog protein plays, highlights the fact that beyond pharmacological molecular determinants, the issue of drug delivery becomes more and more critical [96, 97]. This issue is currently addressed by recent efforts to develop “gemcitabine 2.0.” forms likely to display improved distribution and cellular uptake profiles.

## Gemcitabine prodrugs

In order to overcome various forms of drug resistance and/or to improve drug delivery, recent studies evaluated several gemcitabine prodrugs. Since many clinical studies showed that a low expression of hENT1 was associated with a poor survival of pancreatic cancer patients receiving gemcitabine [97–99], Clavis Pharma developed an elaidic acid prodrug modified at the 5′-sugar position, CP-4126, which was able to bypass hENT1. CP-4126 was similarly effective as gemcitabine in various model systems in vivo and showed an oral efficacy, possibly because CP-4126 could also inhibit CDA, preventing or reducing its first-pass effect [98]. Because of its efficacy in phase II studies, CP-4126 (as CO-101) was tested in a phase II randomized, multicenter trial in comparison with gemcitabine as first-line therapy in metastatic pancreatic cancer patients [99]. Mandatory tumor biopsy specimens were evaluated for their hENT1 expression in both treatment groups. The similar effect of CO-101 and gemcitabine demonstrated an effective conversion of the prodrug to gemcitabine. However, the study did not reach its anticipated endpoint, an increased efficacy of CO-101 compared to gemcitabine in the low hENT1 group of patients. Of note, no difference in survival was found also between the low and high hENT group treated with gemcitabine, suggesting that the role of hENT1 is less important in metastatic disease than after surgery, as shown in the patients in the earlier adjuvant studies. However, it is also possible that the lack of difference between the patients with low and high hENT1 expression was due to a lower specificity of the antibody, which was different from that used in earlier studies.

Another gemcitabine prodrug currently in clinical development is LY2334737, which has valproic acid attached to the N4 of the base. Valproic acid is cleaved off by carboxylesterase 2 (CES2), which is high in the liver and gastrointestinal tract, resulting in an early cleavage of the molecule, as well as in a better antitumor activity in tumors with a high CES2 activity. A phase I dose-finding study with oral administration (daily for 14 days, with 1 week rest) showed linear pharmacokinetics, until the maximal tolerated dose of 40 mg in Caucasian and 30 mg in Japanese patients, with a lower toxicity profile compared to oral gemcitabine [39, 100]. However, using other schedules (every other day for 21 days followed by 1 week rest, or daily for 7 days every other week), the maximal tolerated dose was 90 mg/m^2^, which was recommended for phase II studies for the every other day schedule [101].

Nucana developed another type of prodrug, NUC-1031, which is a gemcitabine analog to which a phosphoramidate ProTide moiety has been added. This novel nucleotide evades all three main cellular resistance mechanisms associated with gemcitabine (i.e., nucleoside transport, dCK-mediated activation and CDA-mediated degradation). NUC-1031 showed activity in cell culture and in in vivo models, including xenografts resistant to systemic gemcitabine treatment, while dFdCTP reaching tenfold higher levels in white blood cells than was found for gemcitabine at similar doses [102, 103]. More recently, NUC-1031 showed clear signs of clinical activity in patients with gynecological cancers. This agent was well tolerated, and a phase Ib study of NUC-1031 in combination with carboplatin is ongoing, while phase III studies are planned in both platinum sensitive and refractory gynecological cancers [104].

Another approach to increase delivery consists in the use nanoparticles that can be designed to allow controlled/sustained drug release [105]. These systems are more stable than liposomes, but retain their low immunogenicity. Gemcitabine-loaded gold nanoparticles targeted to the epidermal growth factor receptor with cetuximab had an increased targeting and activity of gemcitabine in pancreatic tumors in vitro and in mouse tumor models [106]. Similarly, gemcitabine covalently coupled with the natural lipid 1,1′,2-tris-nor-squalenic acid (squalene) at its 4-amino moiety, resulting in 4-(N)-tris-nor-squalenoyl-gemcitabine, which spontaneously assemble into a hexagonal structure with an aqueous core, was active in both human and murine leukemia resistant cell lines and tumors [107].

Gemcitabine can also be covalently coupled via polyethylene glycol (PEG) to another molecule that can target the complex to tumor cells, such as for PEG-gemcitabine conjugates to folic acid, binding specifically to the folate receptors, which are highly overexpressed on the surface of many cancers [108]. Finally, gemcitabine can also be loaded in PEGylated liposomes [109].

## Conclusions and perspectives

There are few drugs in oncology that are as old, but still so widely used as chemotherapeutic targets such as gemcitabine. Gemcitabine is indeed approved and commonly used, alone or in combination, for the treatment of several tumor types, such as NSCLC, pancreatic, bladder, ovarian and breast cancer. In order to improve its antitumor activity while reducing toxic effects, many studies investigated pharmacokinetics [110] and/or the impact of genetic polymorphisms and CDA activity [51, 55], as well as tumor-specific expression of hENT1 mRNA and protein [87, 88], on gemcitabine toxicity and efficacy. These factors appear to be the most promising predictive indicators of outcome in patients receiving gemcitabine chemotherapy [9, 10].

However, most pharmacogenetic studies were retrospective and monocentric, without multiple correction and validation in broader populations. Most phenotypic studies used different methods and specimens; for example, a number of assays have been used to determine CDA activity in various blood compartments [111]. Moreover, most clinical trials on gemcitabine combinations were performed without previous preclinical studies evaluating molecular mechanisms and markers of drug synergistic interaction, while pharmacogenomics studies on tumor specimens did not evaluate tumor heterogeneity and possible evolution of cancer cells after tumor relapse, which should be faithfully documented within multiple samples of the single tumor as well as repeated biopsies.

Therefore, future efforts should be redirected at identifying, both in preclinical models and in the clinical setting, either sensitive or non-responding genotypes or phenotypes. These profiles should be identified with validated methods, which should be used for the appropriate patient enrollment into subsequent prospective studies.

Hopefully, in the near future, the availability of validated genetic/phenotypic platforms will lead to the selection of key factors responsible of the chemosensitivity and toxicity to gemcitabine-based treatments and guide in the choice of more effective rationally based tailor-made treatments for each patient.
